# Predominant risk factors for tick-borne co-infections in hunting dogs from the USA

**DOI:** 10.1186/s13071-020-04118-x

**Published:** 2020-05-13

**Authors:** Kurayi Mahachi, Eric Kontowicz, Bryan Anderson, Angela J. Toepp, Adam Leal Lima, Mandy Larson, Geneva Wilson, Tara Grinnage-Pulley, Carolyne Bennett, Marie Ozanne, Michael Anderson, Hailie Fowler, Molly Parrish, Jill Saucier, Phyllis Tyrrell, Zachary Palmer, Jesse Buch, Ramaswamy Chandrashekar, Breanna Scorza, Grant Brown, Jacob J. Oleson, Christine A. Petersen

**Affiliations:** 1 Department of Epidemiology, College of Public Health, University of Iowa, Iowa City, IA 52242 USA; 2 Center for Emerging Infectious Diseases, University of Iowa Research Park, Coralville, IA 52241 USA; 3 Department of Biostatistics, College of Public Health, University of Iowa, Iowa City, IA 52242 USA; 4 Immunology Program, Carver College of Medicine, University of Iowa, Iowa City, IA 52242 USA; 5 IDEXX Laboratories Inc, One IDEXX Drive, Westbrook, ME 04092 USA; 6 Department of Geographical and Sustainability Sciences, College of Liberal Arts & Sciences, University of Iowa, Iowa City, IA 52242 USA

**Keywords:** Co-infection, Ticks, Dogs, USA, Anaplasmosis, Ehrlichiosis, Babesiosis, Lyme borreliosis

## Abstract

**Background:**

Both incidence and geographical range of tick-borne disease has increased across the USA. Similar to people, dogs are hosts for *Anaplasma* spp., *Babesia* spp., *Ehrlichia* spp. and *Borrelia burgdorferi.* Dogs also share our homes and beds, making them both a sentinel for the ticks in our backyards but also increasing our exposure to ticks. Measures to better track, prevent, and/or treat tick-borne diseases in companion animals can lead to better control and prevention of human tick-borne disease. This study identifies demographic and co-infection risk factors for canine seropositivity to tick-borne infections in a cohort of hunting dogs across the USA.

**Results:**

Human patterns of tick-borne disease co-infection in the USA have been predominantly driven by the geographical distribution of the tick vector. Dogs who tested seropositive for *Anaplasma* spp. were 1.40 times more likely (*P* = 0.0242) to also test seropositive for *Babesia* spp. and *vice versa* (1.60 times more likely, *P* = 0.0014). Dogs living in the West had 5% lower risk (*P* = 0.0001) for *Ehrlichia* spp. seropositivity compared to other regions. Controlling for age and *Anaplasma* spp. seroprevalence, dogs in all three other regions were 2.30 times more likely (*P* = 0.0216) to test seropositive for *B. burgdorferi* than dogs in the West. Dogs seropositive for *B. burgdorferi* were 1.60 times more likely (*P* = 0.0473) to be seropositive for *Anaplasma* spp.

**Conclusions:**

Tick geographical distributions have a prominent impact on the regional distribution of hunting dog exposure to tick-borne diseases. Education concerning regional tick prevalence and disease risk is important for everyone, but particularly dog owners, regarding ticks in their region and protection from infection and co-infection of tick-borne pathogens as they travel or move with their dogs. Dogs are sentinel species for human exposure to ticks, and as such surveillance of canine tick-borne infections and understanding the probability that these infections might be seen together as co-infections helps predict emerging areas where people are more likely to be exposed as well.
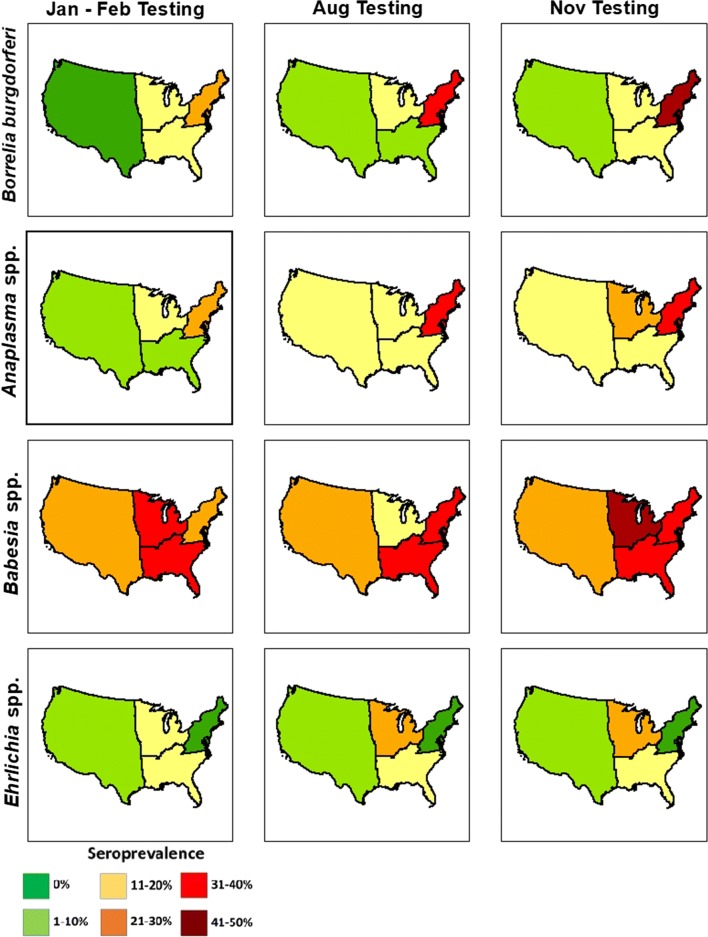

## Background

Ticks transmit a greater variety of pathogens to both people and animals than any other arthropod vector [1]. A recent occupational study found that those who work with hunting dogs compared to those who work in high risk tick environments, were 5.83 times more likely to report having found embedded ticks on their bodies [2]. They found that seropositivity and self-reported diagnosis of Lyme disease were 2.23 times greater in individuals with high tick-environmental exposure [2].

Anaplasmosis, ehrlichiosis, babesiosis and Lyme disease, are tick-borne diseases that pose the greatest threat to human health within the USA. Between 2002–2010, the number of cases of anaplasmosis has risen five-fold [3, 4] from 0.14 cases per 100,000 individuals in 2002 to 0.61 cases per 100,000 individuals in 2010 [5, 6]. In ehrlichiosis-endemic regions, southeastern and south-central USA, incidence rates were as high as 200 cases per 100,000 people [7]. Further, babesiosis recently became a nationally notifiable disease; the reported incidence was 0.8 cases per 100,000 people between 2011–2014 [8, 9]. Lyme disease is the most widely reported vector-borne disease in the USA [10], with 95% of human cases occurring in the Northeast and upper Midwest [11].

Despite the plethora of data associated with Lyme disease in humans, surveillance of Lyme disease and other tick-borne diseases in dogs is less frequent due to a lack of centralized federal surveillance or mandate. Given the close interaction between dogs and people, dogs can serve as an important sentinel species to help track vector-borne disease risks by monitoring trends of infection from tick-borne pathogens in dogs. Using a database from the diagnostic provider IDEXX Laboratories, Bowman et al. [13] evaluated the extent of infection to *Ehrlichia canis*, *Anaplasma phagocytophilum* and *Borrelia burgdorferi* in pet dogs across the USA in 2008. This study found serological evidence of canine infection with these tick-borne pathogens in every USA state. Dogs from the Midwest and Northeast had the highest rates of *B. burgdorferi* and *A. phagocytophilum* seroprevalence while the southern USA had the highest canine *E. canis* seroprevalence. *Ehrlichia* canine seroprevalence was significantly higher than in people (0.2% in endemic areas), at roughly 1–5% [12, 13]. Although previously thought to be rare, the prevalence of *Babesia* spp. in dogs was estimated by the Vector-Borne Disease Diagnostic Laboratory at North Carolina State University to be as high as 21% (*n* = 673 dogs from across the USA) as tested by PCR [14].

This study explored demographic, geographical and biological risk factors for canine seropositivity to *Anaplasma* spp., *Babesia* spp., *Ehrlichia* spp. and *B. burgdorferi* in a cohort of USA hunting dogs. We hypothesized that hunting dogs have higher seroprevalence of tick-borne pathogens compared to pet dogs due to their frequent exposure to tick habitats and lower frequency of tick prevention methods. We investigated how exposure to one pathogen increases the risk of seropositivity to other tick-borne pathogens and how exposure correlates to regionality of tick species.

## Methods

### Study design, enrollment, inclusion criteria

We performed a 12-month longitudinal study to examine to what extent hunting dogs are exposed to tick-borne pathogen infections in the USA and the geographical distribution of these exposures over a year. A total of 214 dogs from 4 different regions (West, Midwest, South and East) in the USA were sampled [15]. Dogs were first tested in January and February 2016, the second sampling period occurred during August 2016, when adult *Dermacentor variabilis*, *Ixodes scapularis* and *Amblyomma americanum* ticks have been shown to be active and feeding on dogs [16–18]. The final sampling period was in November 2016; late tick season. Dogs were enrolled after informed consent from their caretakers and followed protocol as approved by the University of Iowa Institutional Animal Care and Use Committees (IACUC), an AAALAC accredited institution.

Inclusion criteria for dogs in this study were: six months of age or older; not pregnant; up to date on deworming, rabies and distemper multivalent core vaccinations; and not symptomatic for leishmaniosis, Lyme disease, ehrlichiosis, anaplasmosis, or heartworm disease. At enrollment, sex, age, and geographical location were recorded. Licensed veterinarians performed physical exams on each dog and blood was collected from dogs at each time point. Due to the husbandry practices of caring for large numbers (> 20 per location) of cohoused dogs, the dogs enrolled in this study were not provided any routine acaricidal treatments prior to or during this study.

### Sample collection, serology and qPCR

At enrollment, anti-coagulated blood was collected and all dogs were screened serologically using a commercially available multiplexed rapid ELISA (SNAP® 4Dx® Plus Test; IDEXX Laboratories, Inc., Portland, ME, USA) which detects specific antibodies to *A. phagocytophilum*, *A. platys*, *E. canis*, *E. ewingii*, *B. burgdorferi* (*s.l.*) and antigen from *Dirofilaria immitis* [19]. Dogs not meeting the inclusion criterion were excluded.

For the enrolled study cohort, additional serum and EDTA-anticoagulated whole blood samples were collected at each study time point (January, August and November) and more comprehensive serological and molecular diagnostic screening for tick-borne pathogens was performed.

Sera were tested for *Ehrlichia* spp., *E. canis*, *E. ewingii*, *E. chaffeensis*, *Anaplasma* spp., *A. phagocytophilum* and *A. platys* antibodies by synthetic peptide-based ELISA, and for *Babesia* spp. and *Babesia gibsoni* by recombinant protein-based ELISA. Antigens were coated on microtiter plates in 0.05 M sodium carbonate buffer (pH 9.6) and blocked with 2% Tween 20 (Sigma-Aldrich, St. Louis, MO, USA) in 0.1 M Tris buffer (pH 7.4). The plates were incubated with serum samples diluted in sample diluent pH 7.4 (IDEXX Laboratories, Inc.), followed by color development with horseradish peroxidase-conjugated rabbit anti-dog IgG (Jackson ImmunoResearch, West Grove, PA, USA) diluted enzyme conjugate diluent pH 7.4 (IDEXX Laboratories, Inc.) and TMB substrate (Sera Care, Milford, MA, USA). Assay conditions were dependent on antigen and fell within 0.25 to 4.0 µg/ml coating concentration, 1:100 or 1:200 sample dilution, and 1:1000 to 1:4000 anti-species conjugate dilution. Optical density of the resulting color development was measured at 650 nm. Dogs were considered positive if the optical density (OD) was greater than OD cutoffs pre-established based on an independent set of known positive and negative canine samples obtained from globally distributed populations (data not shown) [20]. Seropositivity is presented at species level for initial pathogen temporal tracking purposes. As these pathogens followed similar temporal trends and are often transmitted *via* the same tick species vectors [21], seropositivity was aggregated to the genus level for *Anaplasma* spp., *Ehrlichia* spp., and *Babesia* spp. for co-infection analyses. Dogs were considered exposed if they tested positive *via* ELISA for a given pathogen.

To evaluate the presence of vector borne disease infections, qPCR was performed at a commercial laboratory (IDEXX Reference Laboratories, West Sacramento, CA, USA). Nucleic acid was isolated from the EDTA whole blood samples using the High Pure PCR Template Preparation Kit (Roche Diagnostics, Mannheim, Germany) according to the manufacturer’s instructions. The qPCR assays performed were for the detection of *Ba. canis* (GenBank: AB248735), *Ba. vogeli* (GenBank: EF527401), *Ba. gibsoni* (GenBank: AB248731), *Ba. conradae* (GenBank: AY965739), *Bartonella* spp. (GenBank: L38987), *Rickettsia* spp. (GenBank: AJ293326), *Hepatozoon americanum* (GenBank: AF176836), *H. canis* (GenBank: AF176835), *E. canis* (GenBank: AF403710), *E. chaffeensis* (GenBank: AF403711), *E. ewingii* (GenBank: AY428950), *A. phagocytophilum* (GenBank: DQ519570) and *A. platys* (GenBank: AY848753) nucleic acids. All qPCR testing was performed on the LightCycler 480 instrument (Roche Diagnostics) with data being analyzed with the 2nd derivative maximum method of the instrument software. Samples were considered positive if a crossing point (CP value) was generated. These qPCR results were not used for exposure-based risk factor analyses.

### Statistical analyses

#### Sample size calculation

A power analysis was performed for comparing two independent binomial proportions [22]. Based on the seroprevalence calculated from Bowman et al. [13] using national surveillance data and some cross-sectional data regarding tick-borne disease prevalence in this hunting dog cohort, we needed to enroll 81 total dogs to detect a statistically significant difference between seroprevalence in pet dogs and seroprevalence in our hunting dogs with 80% power at a 0.05 significance level. To detect a clinically relevant change in risk of 0.10 in our analysis we needed 114 dogs to achieve 80% power at a 0.05 significance level. We initially enrolled 214 dogs into the study; 40 dogs (19.16%) were lost to follow-up during the study.

#### Data analysis

Prevalence of *Anaplasma* spp., *Babesia* spp., *Borrelia* spp., and *Ehrlichia* spp. was calculated at enrollment (January-February), August, and November as the number of dogs seropositive for a pathogen divided by the total number of dogs tested at that time point for each respective pathogen.

Descriptive statistics were generated for positive or negative ELISA based on canine sex, age (younger than 6 years or older than 6 years), and geographical region. Our laboratory previously found that the average age of mortality in this hunting dog cohort was six [15]; thus, we categorized age as dogs younger than six years of age *versus* dogs older than six years of age. Chi-square and Fisher’s exact tests were performed to assess associations between demographic factors and tick-borne disease exposure (i.e. age, sex and region) as well as co-infections (i.e. *Anaplasma* spp., *Babesia* spp., *Borrelia* spp. and *Ehrlichia* spp.). Tick-borne disease exposure was classified as being positive at any time for co-infection analyses (*n* = 214). In total we lost 40 dogs to follow-up, and 13 dogs died. Data from dogs lost because of death were continued in the study *via* the last observation carried forward method. We lost 26 dogs between enrollment and August 2016, and 14 dogs between August and November 2016. We performed an analysis to evaluate the missing at random assumption and only found evidence that dogs exposed to *Ehrlichia* spp. were not missing at random. For analysis of region, we made 2 by 4 contingency tables of tick-borne pathogen seropositivity by region (East, West, South and Midwest). The referent group for this analysis was the West, the area with the lowest historical tick-borne pathogen burden. Unadjusted analyses were performed for each tick-borne infection (*Anaplasma* spp., *Babesia* spp., *Borrelia* spp. and *Ehrlichia* spp.) This was followed by two different multiple regression analyses. The first included variables significant in unadjusted analyses. The second analysis was performed using co-exposure variables or pathogens that can be carried by the same tick species, *I. scapularis.* Data are reported as percentage of dogs seropositive and risk ratios were calculated to determine probability of seropositivity. For comparison of pet *vs* hunting dog exposure rates, pet dog exposure rates in Bowman et al. [13] were used. Tick-borne pathogen exposure has changed since 2008, so we assumed in the vacuum of national canine tick-borne disease reporting that a similar trend has occurred for the USA dog population to that reported in people. We extrapolated the potential increase in pet dog tick-borne pathogen exposure rates from rate changes in the US Centers for Disease Control and Prevention human tick-borne disease reporting from 2008 when Bowman et al. [13] was published, to 2016, when our data was collected. We conducted a test of equal proportions between hunting dog and pet dog tick-borne pathogen exposure rates to determine if there were significant differences. All analyses were performed using SAS 9.4 software.

## Results

At the beginning of tick season, 65 dogs (30.37%) tested positive for any tick-borne pathogen on the IDEXX SNAP® 4Dx® Plus Test (*Anaplasma*, *B. burgdorferi* and *Ehrlichia*) and 66 dogs (30.84%) were seropositive to those three tick-borne pathogens *via* ELISA. Of the 66 dogs that tested ELISA-positive to a tick-borne pathogen, 51 (78.46%) dogs were 6 years-old or younger. The highest percentages of seropositive dogs at enrollment were in the East (64.622%) and Midwest (27.69%) regions (Table 1).

**Table 1:** Demographic data of SNAP® 4Dx® Plus Test-positive and ELISA-positive hunting dogs for tick-borne pathogens (*Borrelia burgdorferi*, *Anaplasma* spp., *Babesia* spp. and *Ehrlichia* spp.)

	4Dx SNAP-positive (*n* = 214)	ELISA + (no *Babesia*) (*n* = 214)	ELISA + (with *Babesia*) (*n* = 214)
Positive at enrollment, *n* (% of total)	65 (30.37)	66 (30.84)	97 (45.33)
Sex, *n* (% of positives)			
Male	32 (49.23)	36 (54.55)	51 (52.58)
Female	33 (50.77)	30 (45.45)	46 (47.42)
Age, *n* (% of positives)			
≤ 6 years	51 (78.46)	52 (78.79)	76 (78.35)
> 6 years	14 (21.54)	14 (21.21)	21 (21.65)
Region, *n* (% of positives)			
East	42 (64.62)	46 (69.70)	55 (56.70)
Midwest	18 (27.69)	10 (15.15)	18 (18.56)
South	5 (7.69)	7 (10.61)	12 (12.37)
West	0 (0)	3 (4.55)	12 (12.37)

We were interested in evaluating if there were additional tick-borne infections that could be detected through quantitative polymerase chain reaction (qPCR). Some unique results *via* qPCR from the early tick season were *Ba. conradae* (*n* = 1, East), *Rickettsia* spp. (*n* = 2, East), *H. canis* (*n* = 4, South; *n* = 11, East), *H. americanum* (*n* = 2, South), *E. ewingii* (*n* = 4, Midwest; *n* = 1, South), *A. phagocytophilum* (*n* = 3, East); many of these were co-infections as previously reported [23].

**Fig. 1:**
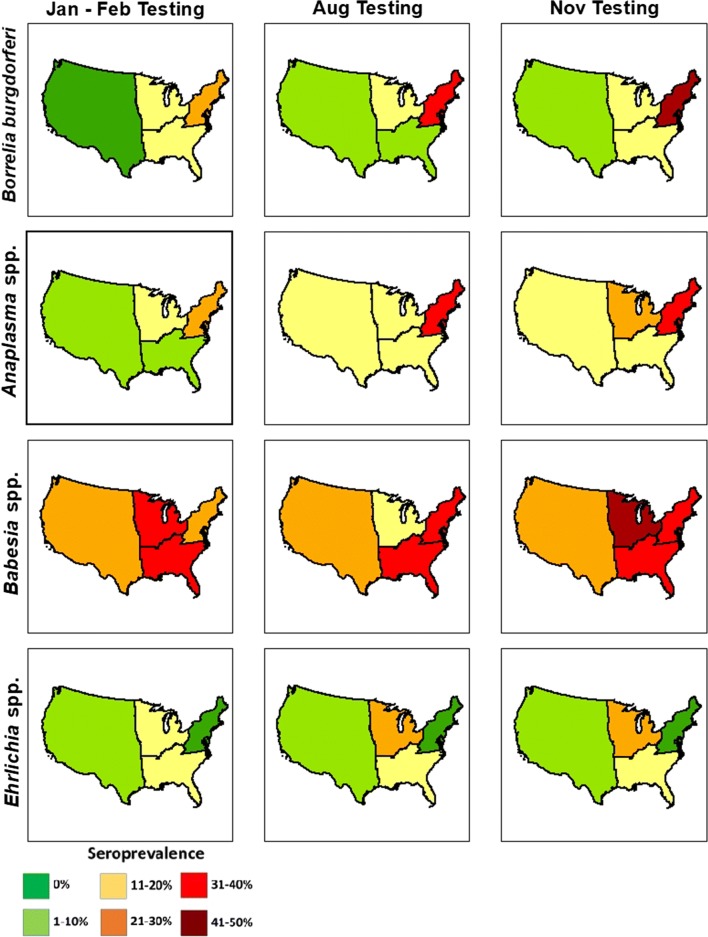
Seroprevalence of tick-borne pathogen exposure was determined *via* ELISA for *B. burgdorferi*, *Anaplasma* spp., *Babesia* spp., and *Ehrlichia* spp. shown as % of regional total dogs tested. Map created in ArcGIS. Sample sizes: January-February testing (*n* = 214); August 2016 testing (*n* = 188); November 2016 testing (*n* = 174)

**Fig. 2:**
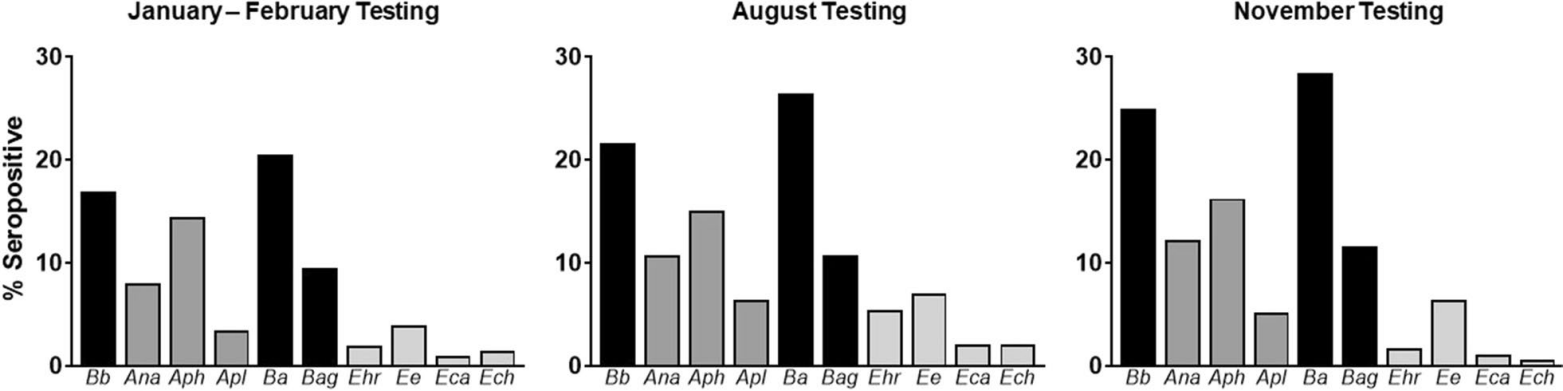
Tick-borne pathogen seropositivity by pathogen species across time. Percent seropositive dogs were calculated from positive ELISA tests from IDEXX laboratories for antibodies to *B. Burgdorferi* (Bb), *Anaplasma* spp. (Ana), *A. Phagocytophilum* (Aph), *A. platys* (Apl), *Babesia* spp. (Ba), *Ba. gibsoni* (Bag), *Ehrlichia* spp. (Ehr), *E. ewingii* (Ee), *E. canis* (Eca) and *E. chaffiensis* (Ech). Sample sizes: January-February testing (*n* = 214); August 2016 testing (*n* = 188); November 2016 testing (*n* = 174)

We evaluated how tick-borne disease seroprevalence occurs regionally over a year using ArcGIS (Fig. 1). *Babesia* spp. seropositivity was found in every region. On average, the Northeast had the highest percent of *B. burgdorferi* (80.00%), *Anaplasma* spp. (67.42%), and *Babesia* spp., (46.96%) seropositive dogs compared to other regions. *Ehrlichia* spp. seroprevalence was highest in the Midwest. We evaluated the change of multiple tick-borne disease seropositivity across the three time points (Fig. 2). Our results show that *Babesia* spp., *Ba. gibsoni*, and *B. burgdorferi* seropositivity remained elevated across all three time points.

**Fig. 3:**
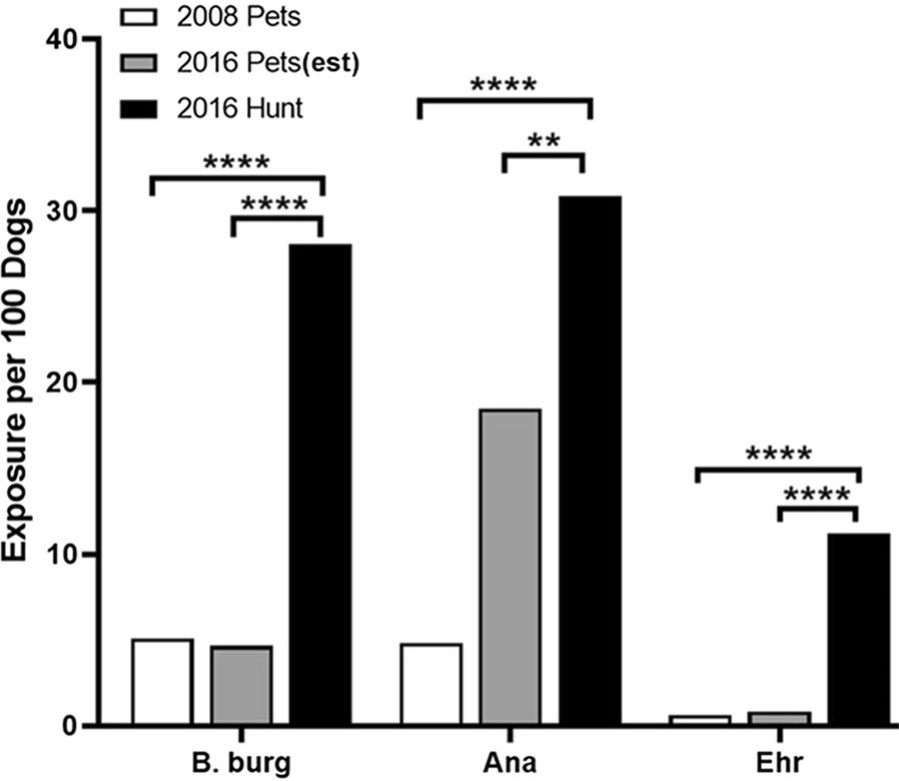
Hunting dogs have significantly higher exposure to tick-borne bacteria compared to companion dogs. Percent positive for each tick-borne pathogen for hunting dogs determined in this study and companion dogs (as reported by Bowman et al. [13]). The adjusted exposure rate for pet dogs in 2016 was calculated using the change in human tick-borne disease exposure between 2008 and 2016 as collected *via* CDC surveillance. The proportion of dogs positive to each tick-borne pathogen for hunting dogs and pet dogs were compared using a test of equal proportions with *P* ≤ 0.05 indicating non-equal differences. ** *P* = 0.005, **** *P* < 0.0001

Bowman et al. [13] previously used national IDEXX SNAP® Test surveillance data compiled over time to determine tick-borne pathogen exposure rates. Here we found that hunting dogs were significantly more exposed to tick-borne pathogens vectored by ixodid tick species when compared to pet dogs in 2008. To account for temporal change in tick-borne disease prevalence between 2008 and 2016, we calculated expected exposure rates for pet dogs based on the change in tick-borne pathogen exposure rates seen in people from surveillance data collected by the CDC. When comparing hunting dog’s exposure to the calculated 2016 pet dog exposure rates, the same significant relationship is present (Fig. 3). Based on these data we wanted to evaluate differences in exposure to each of four pathogens: *B. burgdorferi*, *Anaplasma* spp., *Babesia* spp. and *Ehrlichia* spp.

### Borrelia burgdorferi

The highest percentage of *B. burgdorferi* seropositive dogs came from the East (45.2%), followed by the Midwest (20%). Dogs from the West (4.34%) had the lowest seropositivity (Table 2). Dogs that had *B. burgdorferi* exposure were 1.602 (*P* = 0.0473) times more likely to also be exposed to *Anaplasma* spp.

**Table 2:** *Borrelia burgdorferi* seropositivity risk factors identified through unadjusted analysis

Variable	*B. burgdorferi-*positive	*B. burgdorferi-*negative	Relative risk (95% CI)	*P*-value
Age, *n* (%)			0.9599 (0.5603–1.801)	> 0.9999
≤ 6 years	51 (27.87)	132 (72.13)		
> 6 years	9 (29.03)	22 (70.97)		
Sex, *n* (%)			1.177 (0.7676–1.815)	0.5429
Male	33 (30.28)	76 (69.72)		
Female	27 (24.71)	78 (74.29)		
Region, *n* (%)				< 0.0001*
East	47 (78.33)	57 (37.01)		
Midwest	8 (13.33)	32 (20.78)		
South	3 (5.00)	21 (13.64)		
West	2 (3.33)	44 (28.57)		
% Exposed to *Anaplasma* spp.	25 (37.88)	41 (62.12)	1.602 (1.041–2.421)	0.0473*
% Exposed to *Babesia* spp.	30 (32.26)	63 (67.74)	1.301 (0.8485–1.989)	0.2826
% Exposed to *Ehrlichia* spp.	8 (33.33)	16 (66.67)	1.218 (0.6327–2.083)	0.6299

*Variables significantly associated with *B. burgdorferi* seropositivity at *P* < 0.05

### *Anaplasma* spp

Dogs in the East had the highest rate of *Anaplasma* spp. seropositivity (41.35%) followed by the Midwest (30%). Hunting dogs from the West had the lowest seroprevalence rate (15.2%) (Table 3). Other significant unadjusted risk factors of *Anaplasma* spp. exposure included: sex, *Babesia* spp. co-exposure, and *B. burgdorferi* co-exposure.

**Table 3:** *Anaplasma* spp. seropositivity predicted by sex, region, *Borrelia burgdorferi* and *Babesia* spp. co-exposures through univariate analysis

Variable	*Anaplasma* spp.-positive	*Anaplasma* spp.-negative	Relative risk (95% CI)	*P*-value
Age, *n* (%)			0.847 (0.529–1.482)	0.5353
≤ 6 years	55 (30.05)	128 (69.95)		
> 6 years	11 (35.48)	20 (64.52)		
Sex, *n* (%)			1.58 (1.047–2.411)	0.0379*
Male	41 (37.61)	68 (62.39)		
Female	25 (23.81)	80 (76.19)		
Region, *n* (%)				0.0048*
East	43 (41.35)	61 (58.65)		
Midwest	12 (30.00)	28 (70.00)		
South	4 (16.67)	20 (83.33)		
West	7 (15.22)	39 (84.78)		
*%* Exposed to *B. burgdorferi*	25 (41.67)	35 (58.33)	1.565 (1.039–2.301)	0.0473*
% Exposed to *Babesia* spp.	39 (41.94)	54 (58.06)	1.879 (1.254–2.834)	0.0027*
% Exposed to *Ehrlichia* spp.	11 (45.83)	13 (54.17)	1.583 (0.9239–2.433)	0.1037

*Variables significantly associated with *Anaplasma* spp. seropositivity at *P* < 0.05

### *Babesia* spp

Unlike *B. burgdorferi* and *Anaplasma* spp., the highest number of seroprevalent dogs with *Babesia* spp. was in the Midwest (52.5%) and South (50%) and the lowest numbers in the West (32.6%), with high levels across all regions (Table 4).

**Table 4:** Tick-borne disease seropositivity predicted by age and exposure to *via* unadjusted analysis

Variable	*Babesia* spp.-positive	*Babesia* spp.-negative	Relative risk (95% CI)	*P*-value
Age, *n* (%)			0.6598 (0.49–0.957)	0.0331*
≤ 6 years	74 (40.44)	109 (59.56)		
> 6 years	19 (61.26)	12 (38.71)		
Sex, *n* (%)			1.276 (0.939–1.065)	0.1310
Male	53 (48.62)	56 (51.38)		
Female	40 (38.10)	65 (61.90)		
Region, *n* (%)				0.2665
East	45 (43.27)	59 (56.73)		
Midwest	21 (52.50)	19 (47.50)		
South	12 (50.00)	12 (50.00)		
West	15 (32.61)	31 (67.39)		
% Exposed to *B. burgdorferi*	30 (50.00)	30 (50.00)	1.222 (0.877–1.654)	0.2829
% Exposed to *Anaplasma* spp.	39 (59.09)	27 (40.91)	1.62 (1.199–2.159)	0.0027*
% Exposed to *Ehrlichia* spp.	13 (54.17)	11 (45.83)	1.29 (0.811–1.816)	0.2815

*Variables significantly associated with *Babesia* spp. seropositivity at *P* < 0.05

Despite unexpected regional trends, dogs less than six years of age were 0.6598 (*P* = 0.0331) times less likely to test positive for *Babesia* spp. Dogs positive for *Babesia* spp. were 1.62 (*P* = 0.0027) times more likely to also test positive for *Anaplasma* spp.

### *Ehrlichia* spp

We found the highest exposure to *Ehrlichia* spp. in the Midwest (37.5%) and South (20.83) with the lowest exposure occurring in the West (2.22%) (Table 5). Region was the only significant risk factor for *Ehrlichia* spp. exposure.

**Table 5:** *Ehrlichia* spp. seropositivity predicted by region and age *via* unadjusted analysis

Variable	*Ehrlichia* spp.-positive	*Ehrlichia* spp.-negative	Unadjusted relative risk (95% CI)	*P*-value
Age, *n* (%)			0.4114 (0.196–0.920)	0.0571*
≤ 6 years	17 (9.29)	166 (90.71)		
> 6 years	7 (22.58)	24 (77.42)		
Sex, *n* (%)			1.349 (0.640–2.860)	0.5185
Male	14 (12.84)	95 (87.16)		
Female	10 (9.52)	95 (90.48)		
Region, *n* (%)				
East	3 (2.88)	101 (97.12)		< 0.0001*
Midwest	15 (37.50)	25 (62.60)		
South	5 (20.83)	19 (79.17)		
West	1 (2.17)	45 (97.83)		
% Exposed to *B. burgdorferi*	8 (13.33)	52 (86.67)	1.283 (0.583–2.744)	0.6299
% Exposed to *Anaplasma* spp.	11 (16.67)	55 (83.33)	1.897 (0.905–3.920)	0.1037
% Exposed to *Babesia* spp.	13 (13.98)	80 (86.02)	1.538 (0.734–3.221)	0.2815

*Variables significantly associated with *Ehrlichia* spp. seropositivity at *P* < 0.05

### Multiple regression analyses

To understand how risk factors for tick-borne infections may influence one another, we performed regression analyses using seropositivity for each tick-borne pathogen as the outcome of interest and tested the association of variables identified as significantly associated with exposure to that pathogen from their respective unadjusted analyses (Table 6). We used all significant variables in logistic regression analyses to establish which variables were the most important for the outcome of tick-borne disease co-infection (Table 7). Using only significantly associated variables identified from the unadjusted analysis (Table 2), region was the only variable that remained significant for *B. burgdorferi* exposure. Similarly, region was the only unadjusted variable significantly associated with *Ehrlichia* spp. exposure (Table 5). Western origin was protective (RR = 0.0512; *P* < 0.0001; QIC = 262.7342). Region was not a significant risk factor associated with either *Anaplasma* spp. or *Babesia* spp. exposure on unadjusted analysis (Tables 3, 4). *Anaplasma* and *Babesia* co-exposure were significant risk factors for each other (Tables 3, 4), dogs exposed to *Anaplasma* spp. were 36% more likely to also have been exposed to *Babesia* spp. (RR = 1.3631; *P* = 0.0242; QIC = 578.7200). Conversely, dogs exposed to *Babesia* spp. were 62% more likely to also be exposed to *Anaplasma* spp. (RR = 1.6213; *P* = 0.0014; QIC = 697.3514).

**Table 6:** Pathogens transmitted by ixodid ticks are significant serological co-exposures

	*B. burgdorferi*	*Anaplasma* spp.	*Babesia* spp.	*Ehrlichia* spp.
	Unadjusted RR (95% CI; *P*-value)	Unadjusted RR (95% CI; *P*-value)	Unadjusted RR (95% CI; *P*-value)	Unadjusted RR (95% CI; *P*-value)
Region	– (< 0.0001)*	– (0.0048)*	– (0.2665)	– (< 0.0001)*
Age	0.9599 (0.560–1.801; > 0.9999)	0.847 (0.529–1.482; 0.5353)	0.6598 (0.491–0.957; 0.0331)*	0.4114 (0.196–0.920; 0.0571)*
Sex	1.177 (0.767–1.815; 0.5429)	1.58 (1.047–2.411; 0.0379)*	1.276 (0.9387–1.065; 0.1310)	1.349 (0.6397–2.86; 0.5185)
*B. burgdorferi*	–	1.565 (1.039–2.301; 0.0473)*	1.222 (0.876–1.654; 0.2829)	1.283 (0.583–2.744; 0.6299)
*Anaplasma* spp.	1.602 (1.041–2.421; 0.0473)*	–	1.62 (1.199–2.159; 0.0027)*	1.897 (0.905–3.920; 0.1037)
*Babesia* spp.	1.301 (1.254–2.834; 0.2826)	1.879 (1.254–2.834; 0.0027)*	–	1.538 (0.734–3.221; 0.2815)
*Ehrlichia* spp.	1.218 (0.924–2.433; 0.6299)	1.583 (0.924–2.433; 0.1037)	1.29 (0.811–1.816; 0.2815)	–

*Significant univariate relationship at *P* < 0.05 and included in regressive models

**Table 7:** Adjusted significant predictors for canine tick-borne diseases

Variable	Adjusted RR	95% CI	*P*-value
*Borrelia burgdorferi*			
Region (West referent)	2.2633	1.1274–4.5438	0.0216*
Age (> 6 years-old referent)	1.2514	0.7405–2.2237	0.4444
*Anaplasma* spp. co-exposure	1.4489	0.9891–2.1225	0.0569
*Anaplasma* spp.			
Region (West referent)	1.4329	0.8058–2.5480	0.2207
Age (> 6 years-old referent)	1.1774	0.6943–1.9964	0.5445
Sex (Male referent)	0.7137	0.4588–1.1177	0.1405
*B. burgdorferi* co-exposure	1.3332	0.9199–1.9321	0.1288
*Babesia* spp. co-exposure	1.3631	1.0412–1.7846	0.0242*
*Babesia* spp.			
Age (> 6 years-old referent)	1.0923	0.7181–1.6614	0.6801
*Anaplasma* spp. co-exposure	1.6213	1.2043–2.1826	0.0014*
*Ehrlichia* spp.			
Region (West referent)	0.0512	0.0118–0.2226	< 0.0001*
Age (> 6 years-old *vs* referent)	1.4969	0.6581–3.4044	0.3360

*Variables statistically significantly associated with tick-borne pathogen seropositivity at P < 0.05

We performed multiple logistic regression analysis of tick-borne pathogens transmitted by *I. scapularis* to evaluate risk of co-infection controlling for dog kennel region and age (Table 8). Again, region was the only significant risk factor for *B. burgdorferi* exposure (RR = 2.2601; *P* = 0.0211; QIC = 514.6219). *Anaplasma* spp. exposure and *Babesia* spp. exposure were significant risk factors for each other. In this analysis, dogs exposed to *Anaplasma* spp. were 38% more likely to be co-exposed to *Babesia* spp. (RR = 1.3777; *P* = 0.0186; QIC = 579.5484). Dogs exposed to *Babesia* spp. were 64% more likely to be co-exposed to *Anaplasma* spp. (RR = 1.6384; *P* = 0.0012; QIC = 707.5861).

**Table 8:** Multiple regression analysis of tick-borne pathogens transmitted *via Ixodes scapularis*

Variable	*B. burgdorferi*	*Anaplasma* spp.	*Babesia* spp.
	Adjusted RR (95% CI; *P*-value)	Adjusted RR (95% CI; *P*-value)	Adjusted RR (95% CI; *P*-value)
Region (West *vs* Other)	2.2601 (1.1299–4.5206; 0.0211)*	1.4949 (08402–2.6597; 0.1714)	0.7362 (0.4925–1.1003; 0.153)
Age (> 6 years-old *vs* ≤ 6 years-old)	1.2478 (0.7034–2.2136; 0.4491)	1.2822 (0.7747–2.1223; 0.3336)	1.1129 (0.7446–1.6634; 0.6020)
*B. burgdorferi*	– –	1.3950 (0.9657–2.0152; 0.0761)	1.4160 (0.9917–2.0219; 0.0556)
*Anaplasma* spp.	1.3922 (0.9483–2.0438; 0.0912)	– –	1.6384 (1.2152–2.0880; 0.0012)*
*Babesia* spp.	1.2287 (0.9223–1.6368; 0.1594)	1.3777 (1.0549–1.7991; 0.0186)*	– –

*Variables significantly associated with tick-borne pathogen seropositivity at *P* < 0.05

## Discussion

Dogs are key sentinel hosts for human tick-borne disease exposure. Understanding canine exposure to tick-borne disease improves our understanding of veterinary and human tick-borne exposure. Hunting dog caretakers were at increased risk of finding embedded ticks on themselves compared to people with equivalent outdoor exposures without dogs [19]. This cohort of hunting dogs, much like their caretakers, had significantly higher tick-borne pathogen exposure compared with a cohort of pet dogs in Bowman et al. [13] (Fig. 3). Hunting dogs spend a large amount of time in tick habitats and likely encounter more ticks compared to pet dogs. Further, pet dogs may spend more time in close proximity to people, therefore ticks may be more likely to be noticed and removed. This may limit tick engorgement compared to hunting dogs, reducing the risk of infection, particularly by *B. burgdorferi* that requires 24 hours of attachment to efficiently infect a host [24]. With greater time spent in tick habitats, greater mobility, and longer tick infestation times, it is logical that hunting dogs showed higher frequency of exposure to tick-borne pathogens compared to pet dogs. These characteristics of hunting dogs make them an ideal sentinel host for surveillance of emerging distributions of ticks and the pathogens they transmit.

Geographical region is very closely related to the presence of suitable tick habitat and therefore which ticks dogs encounter [25–27]. We evaluated how regional factors increased the risk of each tick-borne disease. Region was a consistent predictor of exposure to tick-borne diseases. Seropositivity to *B. burgdorferi* and *Anaplasma* spp. was highest in the East while hunting dogs in the more southern parts of the Midwest were seropositive for *Ehrlichia* spp. In addition, we found regional patterns in qPCR findings, with all positive results coming from the South, East, or Midwest regions.

All tick-borne pathogens followed the expected temporal pattern with lower seropositivity in January and February, increasing seropositivity in August and greatest seropositivity in November (Fig. 1). The expected temporal trends for seroprevalence occurred at the species-specific level as well (Fig. 2). Summer humidity and temperatures allow for elevated emergence and activity of nymphs and adult ticks therefore transmission follows the end of summer, depending on the precise emergence and feeding behaviors of each tick species.

Surprisingly, *Babesia* spp., was found in hunting dogs across all regions of the USA. In particular the East, Midwest, and western regions had fairly similar percentages of seropositive dogs. Because the level of *Babesia* spp. exposure found in hunting dogs across the USA was consistently high, another mode of transmission may be involved. Occasional cases of congenital babesiosis in humans have been reported in the USA [28–30]. This route of transmission may be facilitating the spread of *Babesia* spp. outside of *Ixodes* tick habitats in dogs. Travel by hunting dogs may also be involved, but this would be expected to also affect the exposures of all pathogens. Similar to human disease, canine babesiosis is often asymptomatic, is not tested for through the most commonly used veterinary diagnostic test (IDEXX SNAP® 4Dx® Plus Test) and cannot be treated by doxycycline, thus, the true spread of *Babesia* spp. may currently be underestimated, under-reported, and untreated.

Human ectoparasitism by *I. scapularis*, the primary tick vector for *B. burgdorferi*, *Babesia* spp., and *Anaplasma* spp., has been well documented throughout the eastern USA [31]. Our study confirms the likely pattern of *Ixodes* regional transmission, as hunting dogs from the East had greater prevalence of ixodid infections than hunting dogs from any other region. However, *I. scapularis* distribution has recently expanded to include multiple regions across North America [32]. This expansion was mirrored in our results, as we found that hunting dogs exposed to three pathogens transmitted by *I. scapularis* came from multiple geographical regions including the East, Midwest and South, with the East having the greatest prevalence of *B. burgdorferi-* and *Anaplasma*-exposed hunting dogs. Based on the strong association with region observed by three of the four tick-borne pathogens, it is important for healthcare workers to take note of current region and regions veterinary patients and their owners may have visited when considering tick-borne disease diagnoses.

Many of these pathogens can be transmitted by the same tick species; therefore, co-infection is extremely likely, and presence of multiple infections can drastically alter clinical sequelae [33]. Co-exposure to multiple tick-borne pathogens was observed at a high rate within this cohort of hunting dogs. Tick pathogen co-exposures were expected as *B. burgdorferi*, *Babesia* spp. and *Anaplasma* spp. can all be transmitted by *I. scapularis*. Up to 40% of patients with Lyme disease experienced concurrent babesiosis infections [34–38] and 13% experienced concurrent *Anaplasma* infections [34, 38–40]. A high burden of *B. burgdorferi* within *Peromyscus leucopus* (white-footed mouse) was shown to lower ecological thresholds for *Ba. microti* establishment, suggesting individual tick-borne pathogens may enable infection with additional pathogens in host animals [41]. Due to overlapping habitats of *I. scapularis* and *A. americanum*, hunting dogs could be exposed to both vectors; either simultaneously or consecutively [31, 42, 43]. While our analysis did not show significant co-exposures within the *Ehrlichia* spp. seropositive dogs (*n* = 24), we still saw a substantial proportion of *Ehrlichia*-exposed dogs were also exposed to *Anaplasma* spp. (16.67%) and *Babesia* spp. (13.98%). Further loss to follow-up analysis identified a correlation between *Ehrlichia* spp. exposure and being retired or otherwise exiting active hunting status at that kennel indicating that findings particularly regarding *Ehrlichia* infection may be under-representative of the true burden of this specific infection.

Like *I. scapularis*, the geographical range of *A. americanum* is expanding [42, 43]; therefore, co-infections transmitted by these tick species may also be increasing. Such novel interactions may have consequences for human disease. Co-infections, in particular between *B. burgdorferi* and *Babesia* spp., can alter how well a pathogen can be transmitted to ticks [35]. Little is known about how these co-infections may affect transmission in dogs or humans.

## Conclusions

Hunting dogs can be sentinel animals for tick-borne disease exposure to people. People who work with hunting dogs were at elevated risk of tick-borne infection and co-infections. Co-infections can drastically alter how tick-borne diseases clinically present; therefore, healthcare workers, including veterinarians and physicians, may misdiagnose potentially life-threatening tick-borne diseases with increased severity and/or length of clinical manifestations [33, 35, 44–46]. A better understanding of which tick-borne bacterial and parasitic diseases were likely to be found in the USA geographical regions and more likely to be found together, provides important information to inform clinicians of co-infection risk for their area.

## Data Availability

The data supporting the conclusions of this article are included within the article. Our full dataset is available on Research Gate. https://www.researchgate.net/profile/Christine_Petersen/projects.

## Abbreviations

qPCR: Quantitative polymerase chain reaction
ELISA: Enzyme linked immunosorbent assay
